# Regional variations in fluoroquinolone non-susceptibility among *Escherichia coli* bloodstream infections within the Veterans Healthcare Administration

**DOI:** 10.1186/s13756-016-0135-2

**Published:** 2016-10-19

**Authors:** Daniel J. Livorsi, Michihiko Goto, Margaret Carrel, Makoto M. Jones, Jennifer McDanel, Rajeshwari Nair, Bruce Alexander, Brice Beck, Kelly K. Richardson, Eli N. Perencevich

**Affiliations:** 1 Iowa City VA Health Care System, Iowa City, IA USA; 2 Division of Infectious Diseases, Department of Internal Medicine, University of Iowa Carver College of Medicine, 200 Hawkins Drive, Iowa City, IA 52242 USA; 3 Department of Geographical and Sustainability Sciences, College of Liberal Arts and Sciences, University of Iowa, Iowa City, IA USA; 4 Salt Lake City VA Health Care System, Salt Lake City, UT USA; 5 University of Utah School of Medicine, Salt Lake City, UT USA; 6 Department of Epidemiology, College of Public Health, University of Iowa, Iowa City, IA USA; 7 Division of General Internal Medicine, Department of Internal Medicine, University of Iowa Carver College of Medicine, Iowa City, IA USA

**Keywords:** Fluoroquinolones, Antimicrobial resistance, Epidemiology, *Escherichia coli*

## Abstract

**Objectives:**

We sought to define regional variations in fluoroquinolone non-susceptibility (FQ-NS) among bloodstream isolates of *Escherichia coli* across the Veterans Health Administration (VHA) in the United States.

**Methods:**

We analyzed a retrospective cohort of patients managed at 136 VHA hospitals who had a blood culture positive for *E.coli* between 2003 and 2013. Hospitals were classified based on US Census Divisions, and regional variations in FQ-NS were analyzed.

**Results:**

Twenty-four thousand five hundred twenty-three unique *E.coli* bloodstream infections (BSIs) were identified between 2003 and 2013. 53.9 % of these were community-acquired, 30.7 % were healthcare-associated, and 15.4 % were hospital-onset BSIs. The proportion of *E.coli* BSIs with FQ-NS significantly varied across US Census Divisions (*p* < 0.001). During 2003–2013, the proportion of *E.coli* BSIs with FQ-NS was highest in the West South-Central Division (32.7 %) and lowest in the Mountain Division (20.0 %). Multivariable analysis showed that there were universal secular trends towards higher FQ-NS rates (*p* < 0.001) with significant variability of slopes across US Census Divisions (*p* < 0.001).

**Conclusion:**

There has been a universal increase in FQ-NS among *E.coli* BSIs within VHA, but the rate of increase has significantly varied across Census Divisions. The reasons for this variability are unclear. These findings reinforce the importance of using local data to develop and update local antibiograms and antibiotic-prescribing guidelines.

## Introduction

Fluoroquinolones are a synthetic class of antibiotics that have been used in clinical medicine since the 1970s. Fluoroquinolones have excellent oral bioavailability, provide a broad-spectrum of antibacterial activity, and are highly efficacious in the treatment of a variety of infections. However, the overuse of these agents has led to rising rates of FQ non-susceptibility (FQ-NS) [1–3].

Historically, fluoroquinolones have been reliably active against *Escherichia coli*, a common cause of urinary tract infections, bloodstream infections, and intra-abdominal infections. National antibiotic-prescribing guidelines still recommend the empiric use of fluoroquinolones for infections that commonly involve *E.coli* [4, 5], but given the rising prevalence of FQ-NS, the empiric use of fluoroquinolones may no longer be appropriate in some geographic regions [1–3].

In this study, we sought to define regional variations in FQ-NS among bloodstream isolates of *E.coli* in the Veterans Health Administration (VHA) over an 11-year period. Identifying temporal and regional differences in resistance patterns may inform development of national versus regional or local treatment guidelines.

## Methods

We constructed a retrospective cohort of all patients within the VHA who had a blood culture positive for *E.coli* between January 1, 2003 and December 31, 2013. Data from 136 acute care hospitals in 48 US states contributed to the cohort. FQ-NS was defined as a non-susceptible result to at least one FQ: ciprofloxacin, levofloxacin, and/or moxifloxacin. In line with guidelines from the Clinical and Laboratory Standards Institute, only the first isolate was included when the patient had more than one blood culture positive for *E. coli* in the same calendar year [6].

Bloodstream infections (BSIs) were defined as hospital-onset, healthcare-associated, or community-acquired. Hospital-onset BSIs were defined as an *E.coli*-positive blood culture that was obtained after the patient had been hospitalized for ≥48 h. Healthcare-associated BSIs were defined as an *E.coli*-positive blood culture obtained at the time of admission or <48 h of admission if the patient met established criteria for healthcare exposure [7]. Community-acquired BSIs were defined as *E.coli*-positive blood cultures obtained at the time of admission or <48 h of admission in patients who did not meet criteria for healthcare-associated infections.

Hospitals were regionalized based on US Census Divisions (https://www.census.gov/geo/reference/gtc/gtc_census_divreg.html), and annual averages of FQ-NS were mapped. Monthly and annual counts of isolates with and without FQ-NS were measured as outcomes.

Poisson regression models were used to predict the number of isolates with FQ-NS while incorporating total number of isolates as an offset variable. The generalized estimating equations (GEE) method was used to account for grouping effect within Census Divisions. Residual plots were inspected to ensure the appropriateness of all models. An interaction term between time and region was included in the model, and region-specific incidence rate ratios (IRRs) by month and year were estimated to assess variability of slopes across divisions.

## Results

Twenty-four thousand five hundred twenty-three unique *E.coli* (BSIs) were identified between 2003 and 2013 across 136 hospitals and 9 Census Divisions (Table 1). A majority of these BSIs were classified as community-acquired (53.9 %); 30.7 % were healthcare-associated and 15.4 % were hospital-onset BSIs.

**Table 1:** Number of VHA hospitals and *E.coli* bloodstream isolates (BSIs) from each census region, 2003–2013

Census region	Number of hospitals	Number of included *E. coli* BSIs (%)
East North Central	18	2799 (11.4 %)
East South Central	11	2146 (8.8 %)
Middle Atlantic	18	2544 (10.4 %)
Mountain	13	2010 (8.2 %)
New England	7	783 (3.2 %)
Pacific Central	13	3253 (13.3 %)
South Atlantic	26	5358 (21.8 %)
West North Central	16	1896 (7.7 %)
West South Central	14	3734 (15.2 %)

The prevalence of FQ-NS increased with healthcare exposure. The frequency of FQ-NS was 20.2 % for community-acquired *E.coli*-BSIs, 37.0 % for healthcare-associated *E.coli*-BSIs, and 37.7 % for hospital-onset *E.coli*-BSIs.

Throughout the VHA, the percentage of *E.coli*-BSIs with FQ-NS increased from 13.9 % in 2003 to 31.3 % in 2008 and 32.6 % in 2013. Among *E.coli* strains demonstrating FQ-NS, the frequency of non-susceptibility to other antibiotic classes was as follows: 16.6 % extended-spectrum cephalosporins, 34.4 % aminoglycosides, and 0.3 % carbapenems.

**Fig. 1:**
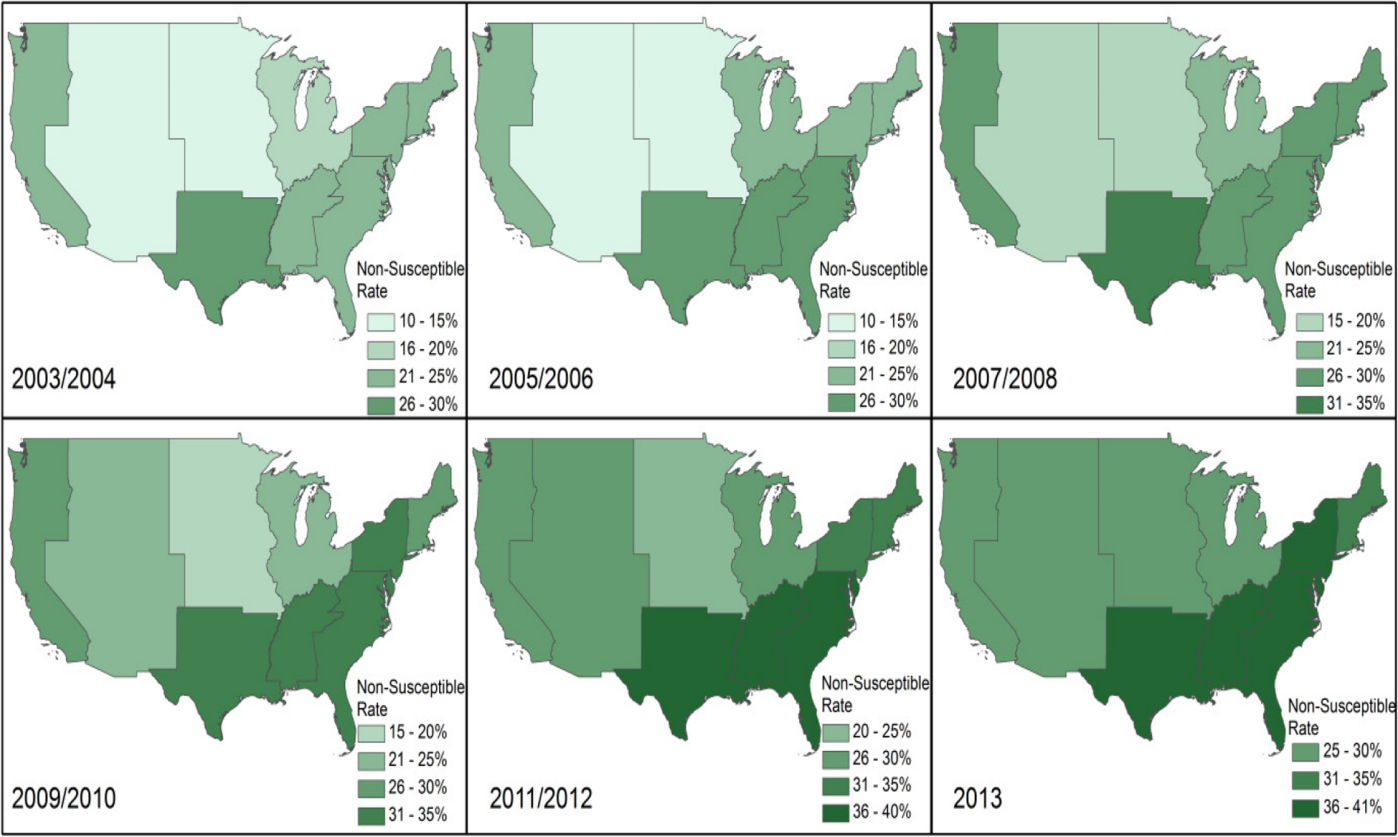
Region-specific changes in FQ-NS among *E.coli* BSIs in the Veterans Health Administration, 2003–2013

The proportion of *E.coli*-BSIs with FQ-NS significantly varied across Census Divisions (range: 20.0–32.7 %; *p* < 0.001). During 2003–2013, the proportion of *E.coli* BSIs with FQ-NS was highest in the West South Central Division (32.7 %) and lowest in the Mountain Division (20.0 %, Fig. 1). In 2013 alone, the highest frequency of FQ-NS among *E.coli*-BSIs was seen in the East South Central Division (39.6 %) and the lowest frequency of FQ-NS was seen in the Pacific Central Division (25.4 %).

**Fig. 2:**
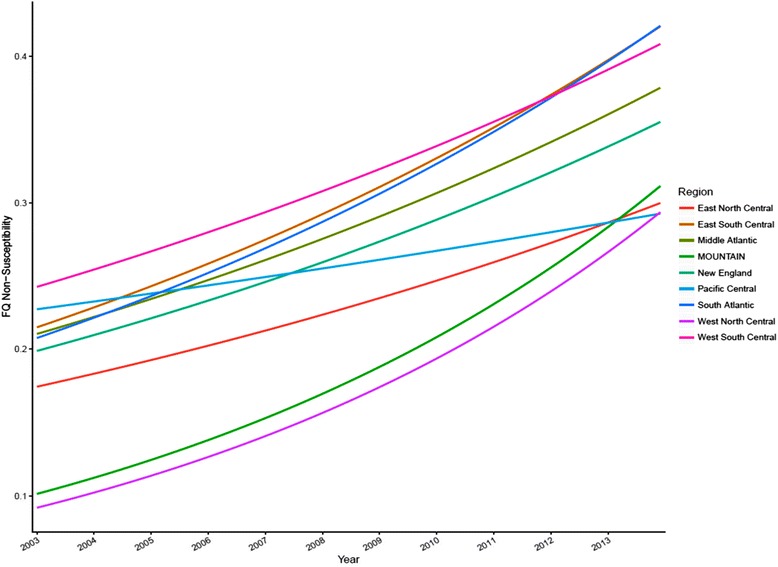
Secular trends in FQ-NS among *E.coli* BSIs within 9 Census Divisions of VHA, 2003–2013

Regression analysis showed that there were universal temporal trends towards higher FQ-NS rates (*p* < 0.001) with significant variability of slopes across Census Divisions (*p* < 0.001, Fig. 2). The annual mean rate of increase in FQ-NS was as follows: East North Central 0.41 % /month; Mountain 0.86 % /month; South Atlantic 0.54 % /month; East South Central 0.51 % /month; New England 0.44 % /month; West North Central 0.89 % /month; Middle Atlantic 0.45 % /month; Pacific Central 0.19 % /month; West South Central 0.40 % /month.

## Discussion

Our study has demonstrated a sustained increase in the frequency of FQ-NS among *E.coli* BSIs across VHA during 2003–2013. The rate of change has significantly varied across Census Divisions. The high prevalence of FQ-NS in some divisions could influence the empiric use of fluoroquinolones for infections thought to involve *E.coli* and suggests that regional or even local guidelines might be preferred over national guidelines.

The clonal expansion of sequence type (ST) 131 *E.coli* is likely a major driver of this rising prevalence of fluoroquinolone resistance [8–11]. ST131 represents one of more than 1000 STs of *E.coli* defined by multilocus sequence typing (MLST). The clonal expansion of ST131 has been a global phenomenon that has not spared the VHA. In a 2011 analysis of *E.coli* clinical isolates from 24 VHA medical centers, ST131 accounted for 78 % of fluoroquinolone-resistant isolates and 28 % of all isolates [12]. The aminoglycoside and carbapenem susceptibility profiles of ST131 strains described in this 2011 study are similar to that of FQ-NS strains in our report. Studies outside of VHA have also found that ST131 accounts for 70–80 % of fluoroquinolone-resistant *E.coli* infections [12–14]. A specific subclone, which represents the vast majority of fluoroquinolone-resistant ST131 isolates, first emerged around 2000 and has since expanded rapidly around the world [15].

The global dissemination of ST131 and its subclones is not well understood. Possible microbiologic contributors include the clone’s enhanced transmissibility, its increased virulence, its resistance to multiple antibiotics, and its success at colonizing the human body [8].

In certain healthcare settings, there is a high colonization pressure with ST131, and this likely facilitates patient-to-patient transmission. Long-term care facilities may serve as reservoirs of this clone [16, 17]. At 2 long-term care facilities in Minnesota, 24 % of residents were colonized with ST131, and molecular analysis demonstrated evidence of intra-facility and inter-facility transmission [17]. Single-center studies at different acute care hospitals found that 50 % of inpatients are carriers [18] and that 13 % of stool samples sent to the microbiology laboratory grew *E.coli* ST131 [19].

Antibiotic-prescribing practices are probably also contributing to the spread of ST131. In a population-based cohort study, multivariable analysis showed that ST131 carriage was predicted by older age, healthcare exposure, and prior antibiotic use [13]. Specifically, prior use of fluoroquinolones, macrolides or extended-spectrum cephalosporins was predictive [13]. Other studies have found that prior fluoroquinolone-exposure is a risk factor for fluoroquinolone-resistant *E.coli*, but these studies did not describe the sequence type of the infecting strains of *E.coli* [2, 20, 21].

It’s unclear why the frequency of FQ-NS significantly varied across Census Divisions within the VHA over time. These differences may reflect broad ecological trends beyond the VHA patient cohort. Alternately, specific infection control and antibiotic-prescribing practices within VHA may be influencing the spread of these strains of *E.coli*. Future research could examine whether use of specific antibiotic agents varies across regions and, if so, whether the variations in antibiotic usage help explain the variability in FQ-NS.

There are several clinical implications to these changing resistance patterns among *E.coli*. Patients undergoing transrectal prostate biopsy typically receive fluoroquionolone prophylaxis. In one study, colonization with fluoroquinolone-resistant *E.coli* was the most important host characteristic associated with infection after transrectal prostate biopsy. Nearly two-thirds of these resistant strains were ST 131 [22]. A survey of Infectious Disease physicians found that there is a perceived increase in the incidence of infections after transrectal prostate biopsies [23]. Furthermore, rising rates of fluoroquinolone-resistant *E.coli* limit options for empiric antibiotic therapy. Among patients with *E.coli* bacteremia, inappropriate empiric antibiotic therapy was associated with worse outcomes in some [24, 25] but not all [26, 27] studies.

Our study has some limitations. First, the cohort included older patients who were predominantly male. As a result, the trends we observed may not be generalizable to other populations. Second, we have argued that ST131 is contributing to the changing epidemiology of *E.coli*, but we did not perform any microbiologic typing. A prior study, however, has demonstrated that ST 131 is prevalent within VHA [12].

## Conclusion

In conclusion, there has been a universal increase in FQ-NS among *E.coli* BSIs within VHA, and the rate of increase has varied across Census Divisions. These findings reinforce the importance of using local data to develop and update local antibiograms and antibiotic-prescribing guidelines.

## Abbreviations

BSI: Bloodstream infection
*E.coli*: *Escherichia coli*
FQ-NS: Fluoroquinolone non-susceptibility
GEE: Generalized estimating equations
VHA: Veterans Health Administration
